# Expression profiling of genes regulated by sphingosine kinase1 signaling in a murine model of hyperoxia induced neonatal bronchopulmonary dysplasia

**DOI:** 10.1186/s12864-017-4048-0

**Published:** 2017-08-29

**Authors:** Viswanathan Natarajan, Alison W. Ha, Yangbasai Dong, Narsa M. Reddy, David L. Ebenezer, Prasad Kanteti, Sekhar P. Reddy, J. Usha Raj, Zhengdeng Lei, Mark Maienschein-Cline, Zarema Arbieva, Anantha Harijith

**Affiliations:** 1 Departments of Medicine, University of Illinois, Chicago, IL 60612 USA; 2 Department of Pharmacology, University of Illinois, Chicago, IL 60612 USA; 3 Department of Biochemistry and Molecular genetics, University of Illinois, Chicago, IL 60612 USA; 4 Department of Center for Research Informatics, University of Illinois at Chicago, Chicago, IL 60612 USA; 5 Department of CoreGenomics Facility, University of Illinois, Chicago, IL 60612 USA; 6 Department of Pediatrics, University of Illinois, Room # 3140, COMRB Building, 909, South Wolcott Avenue, Chicago, IL 60612 USA

**Keywords:** Sphingosine kinase 1, Sphingosine 1 phosphate, Oxidative stress, Lipid signaling, Neonatal lung injury

## Abstract

**Background:**

Sphingosine- 1-Phosphate (S1P) is a bioactive lipid and an intracellular as well as an extracellular signaling molecule. S1P ligand specifically binds to five related cell surface G-protein-coupled receptors (S1P_1-5_). S1P levels are tightly regulated by its synthesis catalyzed by sphingosine kinases (SphKs) 1 & 2 and catabolism by S1P phosphatases, lipid phosphate phosphatases and S1P lyase. We previously reported that knock down of SphK1 (*Sphk1* ^*−/−*^) in a neonatal mouse BPD model conferred significant protection against hyperoxia induced lung injury. To better understand the underlying molecular mechanisms, genome-wide gene expression profiling was performed on mouse lung tissue using Affymetrix MoGene 2.0 array.

**Results:**

Two-way ANOVA analysis was performed and differentially expressed genes under hyperoxia were identified using *Sphk1* ^*−/−*^ mice and their wild type (WT) equivalents. Pathway (PW) enrichment analyses identified several signaling pathways that are likely to play a key role in hyperoxia induced lung injury in the neonates. These included signaling pathways that were anticipated such as those involved in lipid signaling, cell cycle regulation, DNA damage/apoptosis, inflammation/immune response, and cell adhesion/extracellular matrix (ECM) remodeling. We noted hyperoxia induced downregulation of the expression of genes related to mitotic spindle formation in the WT which was not observed in *Sphk1* ^*−/−*^ neonates. Our data clearly suggests a role for SphK1 in neonatal hyperoxic lung injury through elevated inflammation and apoptosis in lung tissue. Further, validation by RT-PCR on 24 differentially expressed genes showed 83% concordance both in terms of fold change and vectorial changes. Our findings are in agreement with previously reported human BPD microarray data and completely support our published in vivo findings. In addition, the data also revealed a significant role for additional unanticipitated signaling pathways involving Wnt and GADD45.

**Conclusion:**

Using SphK1 knockout mice and differential gene expression analysis, we have shown here that S1P/SphK1 signaling plays a key role in promoting hyperoxia induced DNA damage, inflammation, apoptosis and ECM remodeling in neonatal lungs. It also appears to suppress pro-survival cellular responses involved in normal lung development. We therefore propose SphK1 as a therapeutic target for the development drugs to combat BPD.

**Electronic supplementary material:**

The online version of this article (doi:10.1186/s12864-017-4048-0) contains supplementary material, which is available to authorized users.

## Background

Bronchopulmonary dysplasia (BPD) is a chronic condition affecting up to 25% of extreme preterm newborn infants [1, 2]. It is characterized by alveolar simplification, leading to loss of gas exchange area and a decline in lung function that worsens with age [3]. Advances in the care of preterm newborn such as ventilation support has increased the survival rates, however, the practice has also elevated the prevalence of BPD [1]. Though BPD is of multifactorial in origin, exposure to hyperoxia is one of the major contributing factors [4, 5]. There is a preponderance of evidence in support of a role for reactive oxygen species (ROS) induced lung injury in the development of BPD [6].

S1P is a signaling bioactive lipid mediator, generated by phosphorylation of sphingosine catalyzed by two intracellular sphingosine kinases (SphKs) 1 & 2. S1P is transported outside the cell either by ATP binding cassette (ABC) or Spns2 transporter where it exerts its effects through five types of cell surface G-protein-coupled S1P receptors 1-5 (S1PR_1-5_). The pleotropic effects of S1P depend on the pathophysiology and the type of S1P receptor it binds to [7]. S1P is protective in the sepsis model whereas it plays a detrimental role in hyperoxic lung injury and asthma [8]. In lung pathologies such as asthma [8], pulmonary fibrosis [9] and pulmonary hypertension [10, 11], genetic deletion of *Sphk1* or inhibition of SphK1 activity with small molecule inhibitors conferred significant protection from airway/lung inflammation and injury. However, in sepsis-induced lung injury, infusion of S1P protected the animals from lung injury and pulmonary edema by enhancing endothelial barrier function [12]. Thus, S1P is a double edged sword as its action varies depending upon the agent inducing lung injury or the receptor through which it acts.

In our neonatal mouse model of BPD, we showed that hyperoxia increased the expression of SphK1, but not SphK2, leading to increased synthesis of S1P [13]. Deletion of *Sphk1*, but not *Sphk2* gene resulted in decreased S1P levels in lungs accompanied by reduced expression of NADPH oxidase (Nox) 2 & 4 and ROS, leading to amelioration of hyperoxia-induced lung injury. The role of SphK1/S1P on the expression of genes involved in lung inflammation, injury and repair is not clear. We therefore hypothesized that reduced inflammation and improved alveolarization observed in *Sphk1* ^*−/−*^ mice resulted from differential regulation of genes, promoting normal course of lung development while suppressing injury.

To test this hypothesis and to identify the various mechanisms by which the *Sphk1* ^*−/−*^ confers pulmonary protection, differential gene expression analysis in the lungs of *Sphk1* ^*−/−*^ mice before and after treatment with hyperoxia was compared to the Wild Type mice. The results suggest that SphK1/S1P signaling cascade mediates hyperoxia-induced lung injury by modulation of genes related to inflammation, apoptosis, immune responses and redox signaling in mouse lung. Our data also identifies SphK1 as a potential therapeutic target to combat BPD.

## Methods

### Mouse model

#### Mouse experiments and animal care

All experiments using animals were approved by the Institutional Animal Care and Use Committee at the University of Illinois at Chicago (protocol # 15-240). We used male neonatal mice to study the effect of hyperoxia in the newborn developing lungs. The lung development in the neonatal mice at birth is in the late canalicular/early alveolar stage corresponding to that of a preterm human neonate at 24-26 weeks of gestation. *Sphk1* ^*−/−*^ mice were obtained from Dr. Richard L. Proia (NIDDK, National Institutes of Health, Bethesda), and backcrossed to C57BL/6 background for two generations (F2 hybrid). The resultant mixed background of C57BL/6 strain and the original background (F2 hybrid) was used as controls and is referred to hereafter as Wild Type (WT). The WT or *Sphk1* ^*−/−*^ newborn (NB) mice along with the lactating dams were exposed to hyperoxia of 75% O_2_ or normoxia from postnatal (PN) day 1 for 7 days as previously described [13]. NB mice (along with their mothers) were placed in cages in an airtight Plexiglas chamber (55 × 40 × 50 cm), maintained in hyperoxic condition. Two lactating dams were used. Mothers were alternated between hyperoxia and normoxia every 24 h. The litter size was kept limited to 6 pups to control the effects of litter size on nutrition and growth. The animals were maintained as per the University of Illinois protocol for animal use. Oxygen levels were constantly monitored by an oxygen sensor that was connected to a relay switch incorporated into the oxygen supply circuit. The inside of the chamber was kept at atmospheric pressure. The animals were sacrificed and the lung tissues collected, homogenized and whole cell lysates prepared for further analysis, RNA isolation (superior lobe of right lung), and microarray studies.

### Sample processing and microarray based gene expression analyses

We used MoGene 2.0 array (Thermo Fisher Scientific, Waltham, MA) that interrogates over 35 thousand mouse specific RefSeq transcripts and represents a comprehensive platform for the whole transcriptome expression profiling [14].

Lung tissue was perfused with PBS prior to harvesting from the animal, and processed immediately after harvesting. Total RNA was isolated from the whole lung tissue using microRNeasy® kit (Qiagen, Maryland, Cat no. 74004). RNA samples derived from individual animals were separately labeled, hybridized, washed/stained and scanned according to the standard WT PLUS labeling protocol recommended by the manufacturer (Thermo Fisher Scientific, Waltham, MA). Three individual mouse lungs were collected for each of the four experimental groups.

### Data collection

Collected hybridization signals were processed using Genomics Suite 6.6 statistical package (Partek, Inc., Saint Louis, MO). The following parameters were applied for hybridization signal processing: RMA algorithm based background correction, quantile normalization procedure, and probe set summarization [15].

All processed array files were inspected for the following quality metrics: average signal present, signal intensity of species-specific housekeeping genes, relative signal intensities of labeling controls, absolute signal intensities of hybridization controls, and across-array signal distribution plots [16]. All hybridizations passed quality control according to indicated labeling and hybridization controls.

### Identification of differentially expressed transcripts

To identify a subset of genes reactive specifically to the hyperoxic environment depending on the status of *Sphk1* gene, we performed a two-way ANOVA using the level of oxygen and status of *Sphk1* as comparison factors. We compared the following groups: *Sphk1* ^*−/−*^ hyperoxia (*Sphk1* ^*−/−*^ HO), *Sphk1* ^*−/−*^ normoxia (*Sphk1* ^*−/−*^ RA), Wild Type hyperoxia (WT HO) and Wild Type normoxia (WT RA). ANOVA model was based on Method of Moments [17] in combination with Fisher’s Least Significant Difference (LSD) contrast (Tamhane and Dunlop, 2000). The latter allowed calculation of direction and magnitude of change for all pair-wise comparisons between the treatment groups, and was later validated by RT-PCR. Calculated raw *p*-values were corrected for False Discovery Rate (FDR) according to Benjamini-Hochberg (BH) correction procedure [18]. Differentially expressed transcripts were annotated according to Affymetrix NA35 Release of the NetAffx Analysis Center.

### Availability of data and materials

The microarray datasets supporting the conclusions of this article are available in the the National Center for Biotechnology Information Gene Expression Omnibus repository, with unique persistent identifier of NCBI tracking system accession number: 18,084,823, and hyperlink to the datasets is given below.

https://www.ncbi.nlm.nih.gov/geo/query/acc.cgi?acc=GSE87350

### Pathway enrichment analyses and data visualization

We performed pathway enrichment analyses (EA) in order to understand the biological factors driving the protective effect observed in *Sphk1* ^*−/−*^ mice with BPD. Transcripts identified as differentially expressed in KO animals in response to oxygen levels in two-way ANOVA test (FDR cut off of 0.05) were imported into MetaCore Genomic Analyses Tool Release 6.22 (Thomson Reuters) for analyses.

Differentials were analyzed using the “Pathway Maps” ontology and the top 50 most enriched pathways (PW) were identified. The output of the tool contained a substantial number of individual PWs that overlap by genes, representing sub-segment of the same PWs and creating redundancy. In order to reduce duplication, we clustered nodal PWs based on their gene content. We used complete linkage hierarchical clustering on the Jaccard distance between the complete set of genes in each PW, and identified closely related individual entities.

**Fig. 1:**
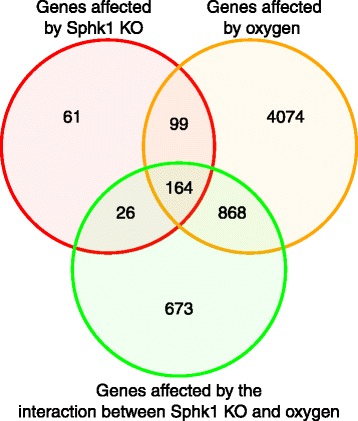
Venn diagram showing the number of genes differentially regulated in the WT and *Sphk1* ^−/−^ neonatal mice exposed to hyperoxia. Two-way ANOVA was performed to analyze the data. There are three variables in the two-way ANOVA performed here such as 1. knock out of *Sphk1* gene, 2. hyperoxia, 3. interaction of *Sphk1* ^*−/−*^ and hyperoxia. Red circle shows all the genes affected by knockout of *Sphk1* gene (61 + 99 + 164 + 26 = 350). *Sphk1* ^*−/−*^ shows 61 genes solely differentially regulated when the corresponding gene was knocked out. Orange circle shows all the genes (4074 + 868 + 99 + 164 = 5205) affected by hyperoxia. 4074 genes were differentially regulated by hyperoxia compared to the corresponding normoxic control unaffected by other factors. Green circle shows genes affected by the interaction between the two factors i.e., *Sphk1* ^*−/−*^ and hyperoxia (673 + 26 + 164 + 868 = 1731). 673 genes were solely affected by the interaction between *Sphk1* ^*−/−*^ and hyperoxia, independent of the direct impact of either

**Fig. 2:**
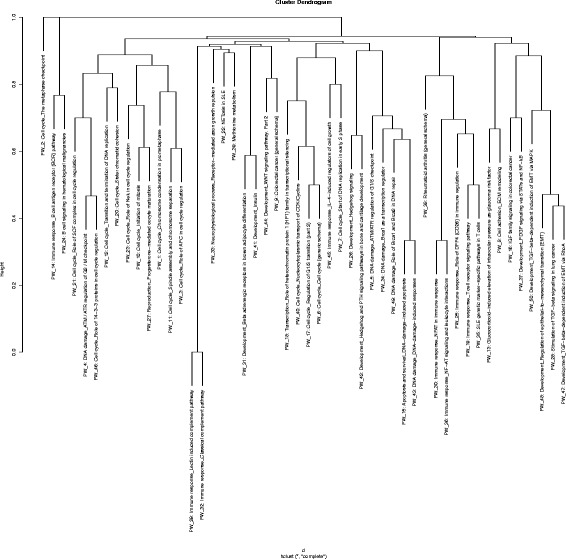
Nodal biological pathways identified as differentially regulated in the animal model of BPD. Differential gene expression analysis was further subjected to pathway enrichment analysis in order to delineate the underlying major biological signaling pathways that could be attributed to the protective effect seen in *Sphk1* ^*−/−*^ mice against hyperoxia induced lung injury. The 7 M clusters of pathways were grouped by similar functions, thus highlighting significant differentially expressed genes most prevalent in our model, as shown here. The pathways combined to form clusters are described here. 1. Cell cycle metaphase check point (cluster 1), 2. DNA damage G2/M check point (cluster 3), 3. DNA-damage-induced apoptosis and survival (cluster 8), 4. Cell adhesion and extracellular matrix remodeling (cluster 10), 5. Pdgf signaling via NF-ĸB pathway (cluster 11a), 6. Wnt signaling and epithelial mesenchymal transition (cluster 11b), and 7. Sphingosine 1 phosphate (S1P) pathway (cluster 12). A detailed description of the pathways and how they were combined to form clusters is given in Additional file 2: Table S2

A measure of the dissimilarity between two PWs (based on their gene sets) scales from 0 to 1; ‘0’ if the sets are exactly the same, and ‘1’ if they are completely different and have no genes in common. For the purpose of biological interpretations, we considered each cluster of closely related PWs as one unit or mega pathway (dissimilarity cut off of 0.6) and combined all associated differential genes for creating heatmaps and analyzing gene interactions as shown in the Venn diagram (Fig. 1) and a dendrogram (Fig. 2).

**Fig. 3:**
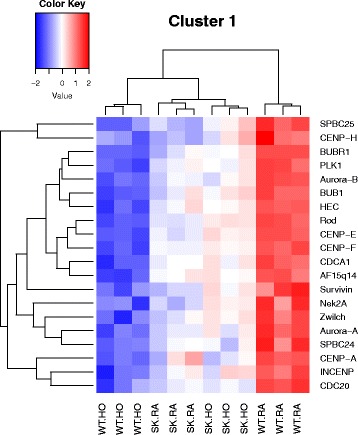
Heatmap showing differentially regulated genes in the nodal pathway related to cell cycle metaphase check point (cluster 1). Differentially expressed genes were analyzed using the “Pathway Maps” ontology and the top 50 most enriched pathways (PW) were identified. We clustered nodal PWs based on their gene content and reduced duplication. Complete linkage hierarchical clustering on the Jaccard distance between the complete set of genes in each PW was done and closely related individual entities were identified. Each cluster of closely related PWs was considered as one unit or mega pathway (dissimilarity cut off of 0.6). All associated differential genes were combined for creating heatmaps and analyzing gene interactions. Clustering of pathways has been detailed in Additional file 2: Table S2. The color key ranging from dark blue to dark red shows the degree of differential regulation from −2 of down regulation or more to +2 of upregulation or more. The color key ranging from dark blue to dark red shows the z-scored normalized expression level. This heatmap depicts the biological nodal pathway showing differential regulation of genes among the 4 different groups such as WT neonatal mice exposed to room air (WT RA), hyperoxia (WT HO), *Sphk1* ^−/−^ mice exposed to room air (SK RA), or *Sphk1* ^−/−^ mice exposed to hyperoxia (SK HO). Selected genes depicted in the heat map is described here. Among the genes prominently downregulated by hyperoxia in the WT include 1) centromere associated proteins, Cenp A, E, F and H, 2) Kinetochore proteins Bubr, Aurora A and B, Bub1 and Zwilch and 3) protein kinases such as Nek2a

**Fig. 4:**
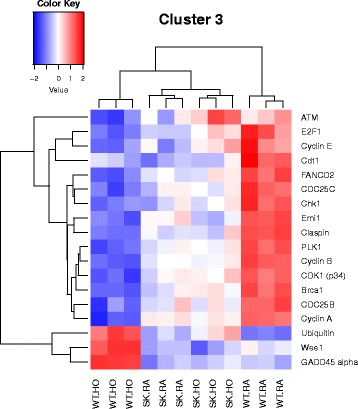
Heatmap showing genes differentially regulated in the nodal pathway related to mostly DNA damage G2/M check point (cluster 3). The cluster combines data from 11 related pathways as shown in Additional file 2: Table S2. Selected genes depicted in the heatmap are described here. Among the genes prominently downregulated by hyperoxia in the WT are 1) Cyclins A, B and D, Cdk1, 2) ATM serine/threonine kinase, Chk1. ATM is activated by DNA double-strand breaks. In contrast, gene for Wee was upregulated in WT HO along with Gadd45 alpha

**Fig. 5:**
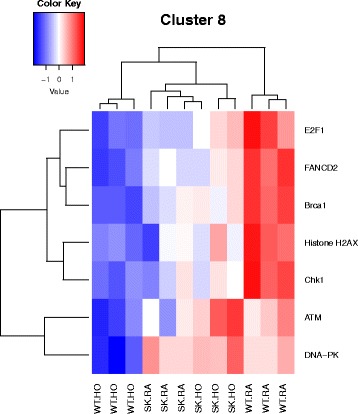
Heatmap showing genes differentially regulated in the nodal pathway related to DNA-damage-induced apoptosis and survival (cluster 8). The cluster combines data from 7 related pathways as shown in Additional file 2: Table S2. Selected genes depicted in the heatmap are described here. Genes related to DNA-damage-induced apoptosis and survival PW are downregulated in the WT HO group. These include Histone 2A component, H2AX contributing to the nucleosome-formation and DNA-PK i.e., DNA-dependent serine/threonine protein kinase

**Fig. 6:**
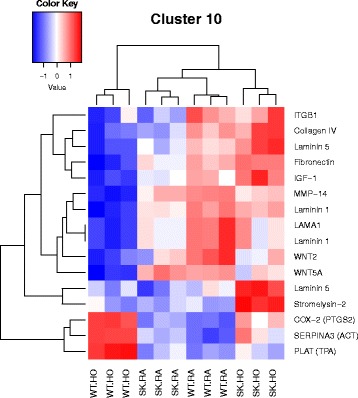
Heatmap showing genes differentially regulated in the nodal pathway related to cluster 10. The cluster combines gene expression analysis data from glucocorticoid-induced elevation of intraocular pressure as glaucoma risk factor and cell adhesion and extracellular matrix remodeling. Among the genes downregulated in WT HO in contrast to *Sphk1* ^*−/−*^ HO are those coding for basal lamina such as Laminin 1, 5 and Collagen IV, matrix metalloproteinases such as MMP-14, MMP-3 (Stromelysin-1) and integrin beta sub unit (ITGB). *Laminin-5* and *Stromelysin-1* are upregulated significantly in *Sphk1* ^*−/−*^ HO. Genes significantly upregulated in WT HO are those coding for *Cox 2, Serpina 3* and *Plat*. Cyclooxygenase (COX) catalyzes formation of prostaglandins such as prostacyclin. *Serpina 3* gene codes for alpha 1-antichymotrypsin which inhibits the activity of proteases, and thereby protects tissues from proteolytic damage. *Sphk1* ^*−/−*^ HO also revealed upregulation of *Cox 2* and *Serpina3* gene expression in response to hyperoxia

To create the heatmaps for selected mega pathways, we plotted z-scored normalized expression levels of differentially expressed genes (FDR < 0.05) across all experimental groups (Figs. 3, 4, 5 and 6). The color key ranging from dark blue to dark red shows the z-scored normalized expression level.

### Realtime RT- PCR validation of microarray results

Total RNA was isolated from mouse lung homogenate using TRIzol® reagent according to the manufacturer’s instructions and purified using the RNeasy® Mini Kit according to the manufacturer’s protocol (Qiagen, MD, USA). iQ SYBR Green Supermix was used to do the real time RT-PCR using iCycler by Bio-Rad, USA. 18S rRNA (sense, 5′-GTAACCCGTTGAACCCCATT-3′, and antisense, 5′- CCATCCAATCGGTAGTAGCG-3′) was used as an external control to normalize expression. Quantitative RT-PCR was performed as previously described [13]. All primers were designed by inspection of the genes of interest and were designed using Beacon Designer 2.1 software. The sequence description of mouse primers used are given in Additional file 1: Table S1. We included negative controls, consisting of reaction mixtures containing all components but for the target RNA, with each of the RT-PCR runs. In order to ensure that amplified products did not represent genomic DNA contamination but the itended mRNA to be amplified, the representative PCR mixtures for each gene were run in the absence of the RT enzyme after first being cycled to 95 °C for 15 min. No PCR products were observed in the absence of reverse transcription,.

Direct comparison of two groups such as WT HO and *Sphk1* ^*−/−*^ HO was done using paired t –test. The level of statistical significance was set at *p* < 0.05.

All the validation studies were done in the exact same cohort used in microarray studies.

## Results

### Outline of the overall transcriptional changes

Figure 1 is a Venn diagram showing the number of genes differentially regulated in the WT and *Sphk1* ^*−/−*^ neonatal mice exposed to hyperoxia based on two-way ANOVA analysis. The diagram depicts number of genes affected in three different categories: 1. *Sphk1* gene knockout, 2. Exposure of the neonatal mouse to hyperoxia, 3. Interaction of *Sphk1* gene knock out and hyperoxia. The intersecting areas represent genes affected by the corresponding condition. The advantage with two-way ANOVA is that the third variable of interaction between the two factors is purely dependent on interaction, and independent of the direct effect of the two factors.

A total of 4074 genes are differentially regulated by hyperoxia compared to corresponding normoxic control. Differential expression of only 61 genes is noted solely due to the impact of *Sphk1* ^*−/−*^ whereas 673 genes were affected by the interaction between *Sphk1* ^*−/−*^ and hyperoxia.

### Pathway enrichment (PW) analyses and elucidation of underlying biological currents

**Fig. 7:**
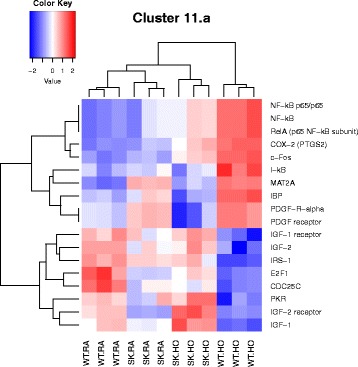
Heatmap showing genes differentially regulated in the nodal pathway related to Pdgf signaling via NF-ĸB (cluster 11 a). NF-κB and related genes (*NF-κB, NF-κB p65, Rel A, I-κB*) are significantly upregulated in WT HO compared to the rest. Genes for insulin like growth factors (IGF) 1 and 2 and their receptors are downregulated in WT HO whereas they are upregulated in *Sphk1* ^*−/−*^ HO. IGF is known to have anabolic effects. *c-Fos* is also noted to be upregulated in WT HO. C-Fos with c-Jun forms AP-1 (Activator Protein-1) complex. AP-1 complex is a transcription factor mediating a wide range of cellular processes such as cell growth, differentiation, and apoptosis

**Fig. 8:**
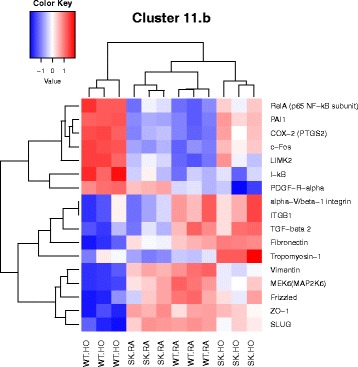
Heatmap showing genes differentially regulated in the nodal pathway related to Wnt signaling and epithelial mesenchymal transition (EMT) (cluster 11 b). The following genes related to EMT were downregulated in WT HO: alpha − V/beta − 1 integrin, fibronectin, vimentin, slug, Zo-1. Frizzled and Wnt signaling were also noted to be downregulated in WT HO. Gene for LIM Domain Kinase 2 (*Limk 2*) was upregulated in WT HO along with other genes described earlier. Limk2 is known to inactivate cofilin and induce formation of stress fibers and focal complexes

**Fig. 9:**
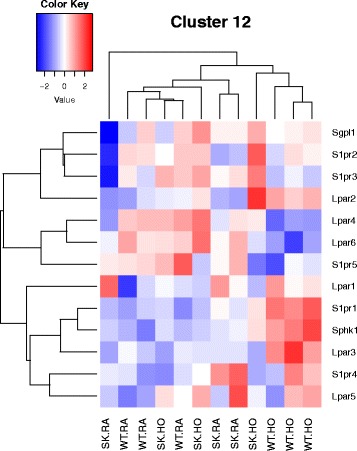
Heatmap representing differentially regulated genes in the WT and *Sphk1* ^*−/−*^ neonatal mice exposed to hyperoxia in pathways related to lipid signaling. *Sphk1*, *S1pr1* and *Lpar3* are some of the genes whose expression is upregulated in WT HO neonatal mice lungs

We identified the top 50 differentially regulated pathways based on the gene expression profiles. Figure 2 depicts a relationship between individual PWs. Additional file 2: Table S2 lists all identified PWs in the order of their position on hierarchical clustering graph, and depicts the clustering of similar pathways. Dissimilarity of 0.6 was used as cut off for the purpose of clustering pathways and reducing the redundancy as described in the Methods. We condensed the data to seven cluster PWs that are differentially regulated among the four groups (WT RA, WT HO, *Sphk1* ^*−/−*^ RA and *Sphk1* ^*−/−*^ HO) studied. Heatmaps show differential expression of selected genes (Figs. 3, 4, 5, 6, 7, 8 and 9).

Most relevant PW clusters are: 1. Cell cycle metaphase check point (cluster 1), 2. DNA damage G2/M check point (cluster 3), 3. DNA-damage-induced apoptosis and survival (cluster 8), 4. Cell adhesion and extracellular matrix remodeling (cluster 10), 5. Pdgf signaling via NF-ĸB pathway (cluster 11a), 6. Wnt signaling and epithelial mesenchymal transition (cluster 11b), and 7. Sphingosine 1 phosphate (S1P) pathway (cluster 12). The differentially expressed genes in the above PWs are illustrated in the heatmaps (Figs. 3, 4, 5, 6, 7, 8 and 9).

### Cell cycle, apoptosis and inflammation

WT HO shows downregulation of the genes essential to maintain normal cell multiplication in complete contrast with *Sphk1* ^*−/−*^ HO and control groups. Centromere proteins (Cenp) play a significant role in assembly of kinetochores and maintenance of mitotic checkpoint signaling [19–21]. *Cenp E, F* and *H* were significantly downregulated in WT HO compared to the rest of the groups. *Bubr1* and *Bub1* genes also play an important role in spindle formation and their expression was downregulated in WT HO whereas their expression was comparable in other groups.

*Bax*, a proapoptotic gene [22] was upregulated 4 fold in WT HO compared to the rest. In contrast, its expression was downregulated in *Sphk1* ^*−/−*^ HO. *Bcl2l*, an antiapoptotic gene, was upregulated 2.9 fold in WT HO compared to the WT RA and elevated 2 fold compared to the *Sphk1* ^*−/−*^ HO.

*p21 (Cdkn1a)* gene expression was increased 7 fold in WT HO and 6 fold in *Sphk1* ^*−/−*^ HO groups compared to normoxia groups. This was accompanied by reduced expression of *Cyclin A*, *B* and *E* genes in the WT HO. Cyclins bind to the dependent kinases, such as the cdk1 protein forming a maturation-promoting factor that facilitates microtubule formation and chromatin remodeling. p21 is a potent cyclin-dependent kinase (Cdk) inhibitor [23, 24]. No difference was noted between the two normoxia groups. In the WT HO, the gene for Wee which inhibits entry of cell into mitosis by inhibiting Cdk1 is upregulated along with *Gadd 45* gene, the family of proteins known to promote inflammation. We noted that multiple genes of *Wnt* signaling pathways including *Frizzled* gene showed a tendency to be downregulated by hyperoxia in the WT compared to the *Sphk1* ^*−/−*^. In contrast, genes related to promotion of inflammation such as *Gadd45 alpha, Cox2, Serpina3, NFkappaB,* and *Pdgf receptor* were upregulated in WT HO group compared to *Sphk1* ^*−/−*^ HO and the rest.

### Sphingosine 1 phosphate signaling pathway

As shown in Fig. 9, there was significant increase in expression of *Sphk1* in the lungs of WT mice exposed to hyperoxia (3.9 fold). In contrast, S1P lyase gene (*Sgpl1*) expression was minimally changed under hyperoxia in the WT. No significant difference was noted in the gene expression of the S1P transporter *Spns2* between various groups. S1P receptor 1 gene (*S1pr1*) was noted to be upregulated in WT HO (2.26 fold) compared to *Sphk1* ^*−/−*^ HO. Interestingly, *S1pr2* expression was relatively lower in *Sphk1* ^−/−^ RA mice that only increased by 1.3 fold upon exposure to hyperoxia. *S1pr3* gene expression showed a tendency to go down in WT HO compared to WT RA. Among the bioactive lipids lysophosphatidic acid (LPA) is an important molecule and is part of the lysophospholipid (LP) family. Lysophosphatidylcholine is converted into LPA by the secreted enzyme, autotaxin. We noted a reduction in autotaxin by 1.9 fold in the WT HO and larger 3.2 fold reduction of this gene expression in *Sphk1* ^*−/−*^ HO. LPA receptors (Lpar) were also differentially expressed in our study. *Lpar2* and *3* were significantly upregulated in WT HO compared to the rest whereas *Lpar4* was downregulated.

Following identification of the PWs, we validated differential expression of specific genes by their biological impact as revealed in our neonatal BPD mouse model, and also by real time RT-PCR.

### Biological impact of *Sphk1* deletion in the animal model correlates with the differential expression of genes

Our animal data correlated strongly with the genes previously identified as differentially expressed in early BPD, and strongly supported the current findings (Table.1). For instance, increased expression of SphK1 mRNA (Fig. 9, Table.1) corresponded to our previous observation that SphK1 protein expression is elevated in neonatal mice exposed to hyperoxia [13, 25]. We have also shown that *Sphk1* ^−/−^ mice exhibited decreased lung S1P levels compared to WT both under normoxia and hyperoxia [25]. IL-6 cytokine level was found to be significantly decreased in the bronchoalveolar lavage (BAL) fluid of *Sphk1* ^*−/−*^ mice compared to the WT exposed simultaneously to hyperoxia [13]. Here, we show that genes of the IL-6 family including its receptors were downregulated in the *Sphk1* ^*−/−*^ HO compared to the WT HO. Increased infiltration of neutrophils in the BAL fluid and lung tissue in the WT HO group was noted compared to *Sphk1* ^*−/−*^ HO and the RA controls. This correlated well with the increased expression of genes related to neutrophil activation such as L, E, P-Selectin and P-Selectin glycoprotein ligand-1 in the WT HO group compared to *Sphk1* ^*−/−*^ HO and the RA controls. Another interesting finding was increased expression of genes coding for proteins involved in DNA damage and apoptosis such as Gadd and Diablo in the WT HO group compared to *Sphk1* ^*−/−*^ HO and the RA controls. This finding correlated well with increased apoptosis observed in the lung tissue of the WT HO group compared to *Sphk1* ^*−/−*^ HO and the RA controls.

**Table 1:** Validation of differential gene expression noted in the microarray by comparing with the biological impact noted in the same animal model

Differential expression of genes in animal model	Biological impact noted in our animal model (Ref 13)
Increased expression of gene *Sphk1* in the WT HO group compared to *Sphk1* ^−/−^ HO and the RA controls.	Increased expression of mRNA and protein of SphK1 in the WT HO group compared to *Sphk1* ^−/−^ HO and the RA controls. Severe lung injury leading to BPD lung morphology in WT HO. Lung injury protection in *Sphk1* ^−/−^ HO.
Increased expression of genesrelated to IL-6 such as Il6, Il6 receptor alpha and Il6 signal transducer in the WT HO group compared to *Sphk1* ^−/−^ HO and the RA controls.	Increased levels of Il-6 measured in the BAL fluid of WT HO group compared to *Sphk1* ^−/−^ HO and the RA controls.
Increased expression of genes related to neutrophil activation such as L, E, P-Selectin and P-Selectin glycoprotein ligand-1 in the WT HO group compared to *Sphk1* ^−/−^ HO and the RA controls.	Increased infiltration of neutrophils in the BAL fluid and lung tissue in the WT HO group compared to *Sphk1* ^−/−^ HO and the RA controls.
Increased expression of genes coding for proteins aggravating DNA damage and apoptosis such as Gadd and Diablo in the WT HO group compared to *Sphk1* ^−/−^ HO and the RA controls.	Increased apoptosis in the lung tissue of the WT HO group compared to *Sphk1* ^−/−^ HO and the RA controls.
Increased expression of genes regulating inflammation in the WT HO compared to *Sphk1* ^−/−^ HO and the RA controls.	Increased cell infiltration and concentration of protein in the BAL fluid of the WT HO group compared to *Sphk1* ^−/−^ HO and the RA controls

### Realtime RT- PCR validation of microarray results

**Fig. 10:**
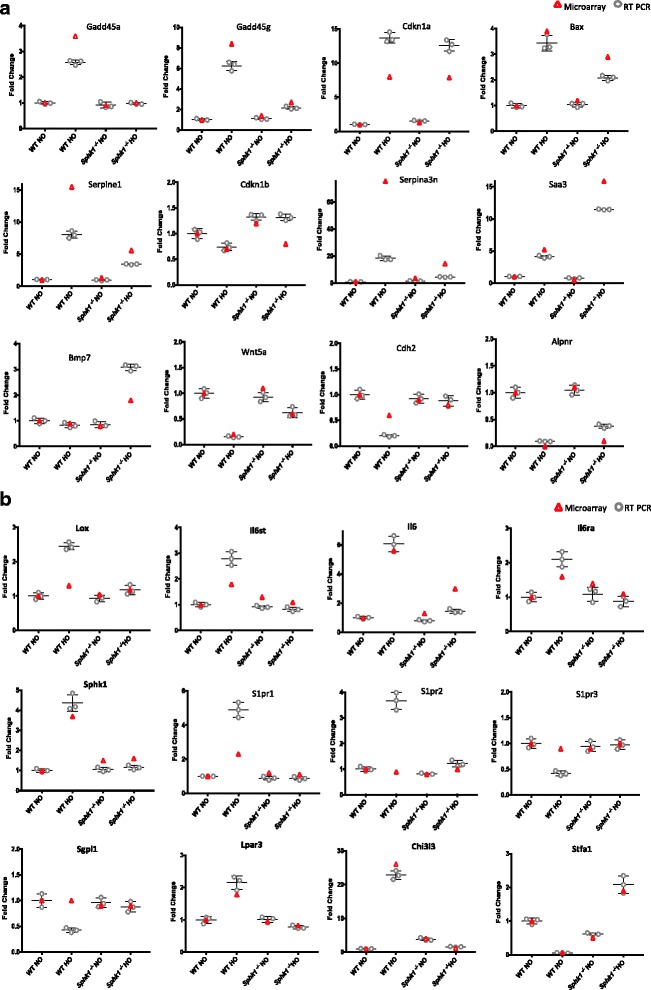
**a** & **b**. Validation of microarray by RT-PCR. The figures represent microarray results in red open triangles and RT-PCR in gray open circles. The four different groups were: WT neonatal mice exposed to normoxia (WT RA), hyperoxia (WT HO), *Sphk1* ^*−/−*^ mice exposed to normoxia (SK RA), or hyperoxia (SK HO). Each graph in the figure represents the corresponding gene studied. The gene names are shown at the top of each individual graph. The validation is evidenced by agreement in vectoral change between microarray and RT-PCR. The fold change may differ between the two methods

Table. 2 and Fig. 10 a & b show realtime RT-PCR performed on select 24 genes based on the observations made from microarray gene expression analysis. Of the 24 genes selected, RT-PCR confirmed differential expression of 21 genes, suggesting an 83% validation. The rationale behind choice of genes for validation is as follows. *Serpina3n*; the most highly upregulated gene in the WT HO group compared to WT RA was an obvious choice for RT-PCR. The most highly upregulated gene in the WT HO group compared to *Sphk1* ^*−/−*^ HO was *Chi3l3*. The most highly downregulated genes common to both HO groups was *Stfa1* [26]. *Aplnr* was noted to be significantly downregulated in both HO groups. *Saa3* was the most highly upregulated gene in *Sphk1* ^−/−^ HO compared to *Sphk1* ^−/−^ RA. These genes were hence chosen as candidates for validation. In addition, *IL-6, IL6st, IL6ra, Gadd45a, Gadd45g, Cdkn1a, Bax, Serpine1, Cdkn1b, Bmp7, Wnt5a, Lox, Cdh2, Sphk1, Sgpl1, S1pr1, S1pr2, S1pr3, and Lpar3,* were chosen from various differentially regulated nodal pathways as they were related to inflammation, cell proliferation and apoptosis, lung development and S1P signaling. More importantly, these genes were also related to biologically relevant findings in our animal model. S1P receptor 2 did not show significant change in the microarray data at gene level, but mRNA showed significant increase in WT HO which was less pronounced in *Sphk1* ^*−/−*^ HO. *Sgpl1* did not show any change in the microarray but WT HO showed significant decrease in the RT-PCR validation. S1P receptor 3 gene in microarray showed a tendency to go down in WT HO which was exaggerated further in the RT-PCR. Though we noted an increased expression of Nox 2 and 4 at both mRNA and protein levels, we did not see a similar increase in the microarray. A brief list of most differentially expressed genes by direct comparison between the four groups of WT RA, WT HO, *Sphk1* ^−/−^ RA and *Sphk1* ^−/−^ HO is given in Additional file 3: Table S3, Additional file 4: Table S4, Additional file 5: Table S5, and Additional file 6: Table S6.

**Table 2:** Microarray data validation by RT-PCR. This table shows a comparison between the differential expression detected by microarray with the corresponding transcript values using RT-PCR. Using RT-PCR, we validated 20/24 genes suggesting 83% concordance between the two methods

		WT RA Fold change	WT HO Fold change	*Sphk1* ^*−/−*^ RA Fold change	*Sphk1* ^*−/−*^ HO Fold change
*Gadd45a*	Microarray Fold Change	1	**3.63168**	0.921811	1.002221
	RTPCR	1	**2.579674**	0.908044	0.967095
*Gadd45g*	Microarray Fold Change	1	**8.38319**	1.43496	2.705904
	RTPCR	1	**6.245498**	1.100627	2.153558
*Cdkn1a*	Microarray Fold Change	1	**7.99461**	1.2607	**7.897151**
	RTPCR	1	**13.72415**	1.537082	**12.61852**
*Bax*	Microarray Fold Change	1	**3.87613**	1.16573	2.869782
	RTPCR	1	**3.43179**	1.032757	2.079701
*Serpine1*	Microarray Fold Change	1	**15.4903**	1.32755	5.559448
	RTPCR	1	**8.024327**	0.94901	3.421921
*Cdkn1b*	Microarray Fold Change	1	0.708453	**1.16238**	0.75834
	RTPCR	1	0.740789	**1.324503**	**1.318684**
*Serpina3n*	Microarray Fold Change	1	**75.388**	**3.72853**	**14.3611**
	RTPCR	1	**18.49097**	**1.648514**	**4.635865**
*Saa3*	Microarray Fold Change	1	5.19527	0.517552	**15.89733**
	RTPCR	1	4.095082	0.74759	**11.46964**
*Bmp7*	Microarray Fold Change	1	0.907484	0.817163	**1.759001**
	RTPCR	1	0.821678	0.857167	**3.075663**
*Wnt5a*	Microarray Fold Change	1	0.2359	**1.07608**	**0.638945**
	RTPCR	1	0.152372	**0.926221**	**0.623315**
*Cdh2*	Microarray Fold Change	1	0.642012	**0.879343**	**0.764438**
	RTPCR	1	0.199199	**0.924477**	**0.879041**
*Aplnr*	Microarray Fold Change	1	0.049004	**1.06134**	**0.147246**
	RTPCR	1	0.092198	**1.04678**	**0.375555**
*S1pr1*	Microarray Fold Change	1	**2.26942**	1.17521	1.116978
	RTPCR	1	**4.894727**	0.889052	0.87604
*S1pr2*	Microarray Fold Change	1	**0.921563**	0.750461	**0.997753**
	RTPCR	1	**3.656166**	0.81154	**1.212284**
*S1pr3*	Microarray Fold Change	1	**0.933923**	**0.936387**	**1.03291**
	RTPCR	**1**	0.415816	**0.944109**	**0.978297**
*Lox*	Microarray Fold Change	1	**1.30887**	1.04026	1.181007
	RTPCR	1	**2.450447**	0.932973	1.175259
*Il6st*	Microarray Fold Change	1	**1.79941**	1.29187	1.072552
	RTPCR	1	**2.79062**	0.905679	0.820019
*Il6*	Microarray Fold Change	1	**5.55994**	1.29187	3.028182
	RTPCR	1	**6.072546**	0.791731	1.45494
*Il6ra*	Microarray Fold Change	1	**1.64621**	**1.40755**	1.136742
	RTPCR	1	**2.101202**	**1.082853**	0.876843
*Sgpl1*	Microarray Fold Change	1	**0.997442**	**0.890375**	**0.938402**
	RTPCR	1	0.424413	**0.963454**	**0.875726**
*Sphk1*	Microarray Fold Change	1	**3.70231**	1.51325	1.556574
	RTPCR	1	**4.364135**	1.047004	1.149245
*Lpar3*	Microarray Fold Change	1	**1.78**	0.95	0.83
	RTPCR	1	**2.153558**	1.008994	0.773786
*Chi3l3*	Microarray Fold Change	1	**26.0615**	4.07383	1.2288
	RTPCR	1	**22.807**	3.806	1.5576
*Stfa1*	Microarray Fold Change	1	**0.0663964**	0.505696	**1.89924**
	RTPCR	1	**0.0537**	.62	**2.086**

Bold Differentially regulated gene expression

## Discussion

Neonatal mice exposed to hyperoxia reveal all the major pathological features of human BPD, and therefore serve as an excellent model to investigate the underlying pathologies and to identify potential therapeutic targets [27, 28]. S1P has been shown to play dual roles, both protective as well as pathological, depending upon the clinical, i.e., pathological situations [13, 29, 30]. It is currently known that in asthma, S1P induces degranulation of mast cells and promotes adverse remodeling response of airway smooth muscle cells [31, 32]. Inhibition of S1P receptors using Fingolimod serves as the only effective therapy against multiple sclerosis [33]. However, it has also has been shown that S1P promotes endothelial integrity in sepsis model of endothelial injury [30]. In this background, we showed in our previous study using *Sphk1* ^*−/−*^ neonatal mice that S1P/SphK1 promotes hyperoxic lung injury by promoting formation of ROS [13]. ROS production was mediated through S1P_1_ and S1P_2_ receptor signaling [25]. The opposing effects of SphK1 and SphK2 also have been described earlier [34]. The two enzymes are located in different compartments of the cell, and the production of localized S1P has distinct functions. SphK1 has been shown to be predominantly cytosolic whereas SphK2 is considered a nuclear protein [35]. Deletion of SphK1 results in about 50% reduction in serum levels of S1P whereas deletion of SphK2 results in only about 25% reduction of S1P [25, 36]. The reason for this is not known though the production of S1P can be compensated by one enzyme in the absence of the other. Under normoxia, there is no morphological difference or difference in life span between WT and *Sphk1* ^*−/−*^ mice. S1P is essential for normal lung development as it plays a significant role in angiogenesis essential for alveolar formation [37]. In addition, even complete knock out of SphK 1 enzyme resulted in only 50% reduction of S1P levels as SphK 2 compansates for the loss of SphK1. The difference is exaggerated only under pathological conditions such hyperoxia when an increase in SphK1 enzyme expression is prevented in *Sphk1* ^*−/−*^ mice. This could be the reason for similarity of gene expression between WT and *Sphk1* ^*−/−*^ at baseline normoxia. Under hyperoxia, an elevated expression of SphK 1 leads to increased production of S1P followed by reduced ROS production in the lungs. Deletion of SphK1 also attenuated inflammation and apoptosis under hyperoxia. In order to further understand the key players in hyperoxic lung injury in neonates, we applied microarray based differential gene expression analyses. In general, we observed a strong agreement between our previous in vivo findings in the neonatal mouse BPD model and specific differential gene expression profiles revealed in the present study and the data is summarized in Table 1.

Several novel findings that logically fit into the pathology of BPD emerged from the current study. Centromere proteins Cenp E, F and H were significantly downregulated in WT HO compared to the rest of the groups. Cenp E appears during prometaphase, Cenp F in the late anaphase and telophase whereas Cenp H in both interphase and metaphase [38, 39]. Downregulation of these proteins that play a crucial role in the assembly of kinetochores and spindle elongation will invariably dampen cell proliferation leading to the arrest of organ growth [40, 41]. Another example is the case with *Bub1* and *Aurora B* kinase genes which were downregulated in WT HO but not so in *Sphk1* ^*−/−*^ HO. Bub1 and Aurora B kinase work in tandem, promoting formation of stable bipolar kinetochore-microtubule attachments [42]. Both Bub1 and Aurora B kinase are recruited to kinetochores independent of each other but have an additive effect when depleted simultaneously. Bubr1 stabilizes kinetochore–microtubule attachment and helps chromosome alignment by associating with unattached/incorrectly attached kinetochores [42]. Bubr1 forms part of the mitotic checkpoint complex (MCC) that also contains Bub3*.* Bub 1 depletion leads to the accumulation of misaligned chromatids [42]. It is very likely that the above kinetochore related genes will influence cell division and proliferation, and thus indirectly may contribute to elevated apoptosis seen in hyperoxia induced neonatal lung damage, and needs further investigation.

Interestingly, increased *p21 (Cdkn1a)* gene expression was observed in both WT HO and *Sphk1* ^*−/−*^ HO groups compared to normoxia groups. This is consistent with previous studies in mammalian and human cells exposed to hyperoxia that show a block in the G1 to S phase progression mediated by an increase in Cdk inhibitor p21WAF1/CIP1 within the first 12 h [23, 24, 43]. Cell cycle arrest is essential for the repair of hyperoxia induced tissue damage, and to avoid the replication and propagation of cells that harbor potentially hazardous mutations [44, 45]. The role of Cdk inhibitor p21 in causing cell cycle arrest is controversial. On one hand, it has been shown to act as an inhibitor of apoptosis while on the other hand, it has also been shown to induce cell proliferation [46]. The differences in response to hyperoxia seen in WT HO and *Sphk1* ^*−/−*^ HO are hence unlikely to be mediated through p21.

Despite an increased expression of p21, there was preserved alveolarization in the *Sphk1* ^*−/−*^ HO group compared to WT HO. This is supported by the current observation that Wnt signaling pathways were better preserved in the *Sphk1* ^*−/−*^ HO group compared to WT HO. Wnt/β-catenin1 pathway is known to play a critical role in normal lung development [47]. In contrast, expression of promoters of inflammation such as *Gadd45 alpha, Cox2, Serpina3* and *NFkappaB* were upregulated in WT HO group compared to *Sphk1* ^*−/−*^ HO. The present findings are also in agreement with our observation made in the lungs of adult mice exposed to hyperoxia [25]. Gadd45 and Gadd153 mRNAs were detected respectively after 48 and 72 h of exposure of adult mice to hyperoxia [48]. In situ hybridization and immunohistochemistry studies showed that in hyperoxia Gadd45 and Gadd153 expression were increased in the bronchiolar epithelium and type II alveolar cells, respectively.

Cell cycle arrest is known to trigger apoptosis. In our previous study, we noted a clear increase in apoptosis in the lungs of WT neonatal mice exposed to hyperoxia [25]. Our current findings are in accordance with the above finding. *Bcl-XL (Bcl2l),* an antiapoptotic gene, was upregulated 2 fold in WT HO compared to *Sphk1* ^*−/−*^ HO but more interestingly, *Bax* which is a proapoptotic gene was upregulated 4 fold in WT HO compared to the rest. The gene was downregulated in *Sphk1* ^*−/−*^ compared to the WT HO. Studies have shown that increases of the antiapoptotic gene *Bcl-XL* were counterbalanced by similar increases of the proapoptotic gene *Bax* [49]. In our study, we see an increase in proapoptotic gene in both WT and *Sphk1* ^*−/−*^ groups exposed to HO but rise in proapoptotic as well as antiapoptotic genes was less pronounced in *Sphk1* ^*−/−*^ compared to WT exposed to HO.

Delving further into lipid mediated signaling other than S1P, we noted a significant increase in gene expression of LPA receptor 3 in the WT HO (1.78 fold increase with FDR of 0.014869) which is in agreement with hyperoxia studies carried out in rat BPD models [50]. Related studies in humans and rats revealed increased protein expression of autotaxin, Lpar1 and Lpar3 that co-localized to airway epithelial cells following exposure to hyperoxia [51]. These proteins were significantly distributed in vascular endothelial and mesenchymal cells during the developmental phase of the lung in the first postnatal week. It will be interesting to see the distribution of LPA receptors in lung epithelium of neonatal mice exposed to normoxia and hyperoxia.

Human airway epithelial cell lines, including BEAS-2B, NCI-H292, and A549, increase their intracellular glutathione levels within hours in response to hyperoxia in vitro [52, 53]. The underlying mechanism could be due to elevated levels of glutamylcysteine ligase that is essential for novo synthesis of glutathione; however, we did not see any change in expression of glutamylcysteine ligase following exposure to hyperoxia.

Though we noticed 83% correlation between microarray and RT-PCR, there was lack of correlation noted in 17%. The disparity in expression between gene and mRNA could be due to the following factors. Microarray data reflects the total expression of the whole genome studied whereas mRNA expression by RT-PCR reflects the changes in a particular part of the genome studied by the primers selected. In addition, the mRNA transcribed would undergo post-transcriptional modification to form the final mRNA which undergoes translation.

We also compared the current differential gene expression data in response to hyperoxia in the neonatal mouse BPD model to the human data on BPD (Table 3). The BPD in humans is classified into ‘Early’ and ‘Late’ gestation by the authors [54]. ‘Early’ gestation was defined as estimated gestational age (EGA) of <27 wk. at birth and <35 wk. EGA at death. ‘Late’ gestation was defined as estimated gestational age of >27 wk. at birth and >35 wk. EGA at death. A total of 159 genes were significantly affected in the BPD lung tissue as compared to the controls in humans [54]. 36 of these genes that were significantly regulated were common between the present study and the human data. Out of the 36 genes, 11 correlated with the differential gene regulation in our animal model. Among those genes, we noted that the expression of human genes *IL1RL1, SFN, SH3RF2* and *SELE* was significantly elevated in human BPD as well as in murine WT HO. *CD177, ALDH1A3, CXCL5*, and *MARCO* gene expression was significantly elevated in the lungs of BPD patients as well as in WT HO and *Sphk1* ^*−/−*^ HO groups, suggesting that these responses to hyperoxia were not mediated through SphK1. The fact that expression of murine genes *Sh3rf2, Sele and Ch25h* showed significant elevation in WT HO group compared to *Sphk1* ^*−/−*^ HO group suggests that the WT neonatal mice exposed to hyperoxia closely resemble human BPD. It is thus possible that these three genes could be mediated through SphK1/S1P pathway. In conclusion, the striking similarities in the differential expression of specific genes between human BPD and neonatal mice exposed to hyperoxia suggests that the neonatal mouse model investigated here resembles ‘Early’ gestation BPD.

**Table 3:** Comparison of the current differential gene expression analysis to published human BPD microarray data. Human lung microarray had four groups such as early gestation control, late gestation control, early BPD and late BPD. Genes found to be differentially regulated in response to hyperoxia that are common to human and mouse neonatal lungs are presented here

Gene Symbol (Gene name)	Early Gestation Control Lungs	Late Gestation Control Lungs	Early Gestation Age BPD	Late Gestation Age BPD	WT HO Vs. RA (BPD equivalent)	SK HO Vs. RA (BPD protection)	Gene function
CD177 (CD177 Molecule)	↓	↓	↓	**↑**	**↑ 2.26378**	**↑ 2.60461**	Neutrophil activation, transmigration
IL1RL1(interleukin 1 receptor like 1)	↓	↓	**↑**	**↑**	**↑ 1.5**	No change	Mediates cytokine-induced immune and inflammatory responses.
SFN (14-3-3 protein sigma)	↓	↓	**↑**	**↑**	**↑ 1.5**	No change	Promotes p53-regulated inhibitor of G2/M progression
SH3RF2(SH3 Domain Containing Ring Finger 2)	↓	↓	**↑↑**	**↑**	**↑↑ 4.8**	**↑ 1.58**	Protein ubiquitination
NNMT(Nicotinamide N-Methyltransferase)	↓	↓	**↑**	**↑**	No change	↓ 1.71	N-methylation of pyridines to form pyridinium ions
LIPG (Lipase G, Endothelial Type)	↓	**↑**	**↑↑**	**↑**	No change	↓ 3.29	Molecular bridge between endothelial cells and lipoproteins.
SELE (Selectin- E)	↓↓	↓	**↑↑**	**↑**	**↑ 2.28**	**↑ 1.3**	Recruits leukocytes to the site of injury
ALDH1A3 (Aldehyde Dehydrogenase 1 Family Member A3)	↓↓	↓↓	**↑↑**	**↑**	**↑ 1.9**	**↑ 2.03**	Detoxification of aldehydes
CH25H (Cholesterol 25-Hydroxylase)	↓↓	↓↓	**↑↑**	**↑↑**	**↑↑ 22.9**	**↑ 1.6**	Catalyze the hydroxylation of hydrophobic substrates.
CXCL5 (CXCL5 or ENA78)	↓↓	↓↓	**↑**	**↑↑**	**↑ 3.57**	**↑ 3.26**	Recruit neutrophils, promote angiogenesis and remodel connective tissues
MARCO (macrophage receptor with collagenous structure)	↓↓	↓	**↑↑**	**↑↑**	**↑ 2.18**	**↑ 2.45**	Part of the innate antimicrobial immune system

Up arrow indicates increase in gene expression, down arrow indicates decrease in gene expression. Increased expression symbol bolded as well

## Conclusion

In summary, this study provides the first ever genome-wide analysis of differential expression in the lungs of neonatal BPD model as it relates to SphK1/S1P signaling. WT HO was associated with significantly increased expression of genes that promote apoptosis, retard cell multiplication, inflammation and extracellular matrix remodeling, whereas the *Sphk1* ^*−/−*^ mice exposed to hyperoxia showed a downregulation of the corresponding genes. Interestingly, the differential gene regulation in our animal model of BPD shows similarity with ‘Early’ gestation BPD. Our study signifies potential role of SphK1/S1P signaling in the pathogenesis of BPD, and thereby makes an attractive therapeutic target for future drug development.

## Additional files

Additional file 1: Table S1. Sequences of sybr green mouse primers used for RT-PCR. (DOCX 38 kb)

Additional file 2: Table S2. Top fifty enriched pathways are listed here by cluster. The process of clustering is explained in the materials and methods. (XLSX 20 kb)

Additional file 3: Table S3. Comparison of the 10 most highly differentiated genes between WT HO and *Sphk1* ^*−/−*^ HO groups. (XLSX 10 kb)

Additional file 4: Table S4. Comparison of the 10 most highly differentially expressed genes between WT RA and *Sphk1* ^*−/−*^ RA groups. (XLSX 9 kb)

Additional file 5: Table S5. Comparison of 10 most highly differentially expressed genes between *Sphk1* ^*−/−*^ RA and *Sphk1* ^*−/−*^ HO groups. (XLSX 31 kb)

Additional file 6: Table S6. Comparison of 10 most highly differentiated genes between WT RA and WT HO groups. (XLSX 9 kb)

## Abbreviations

ABC: ATP binding cassette
ANOVA: Analysis of variance
BAL: Bronchoalveolar lavage
BPD: Bronchopulmonary dysplasia
CDK: Cyclin dependent kinase
Cenp: Centromere protein
ECM: Extra cellular matrix
EGA: Estimated gestational age
FDR: False discovery rate
GADD: Growth arrest and DNA damage
HO: Hyperoxia
KO: Knockout
LPA: Lysophosphatidic acid
MCC: Mitotic checkpoint complex
NB: Newborn
NO: Normoxia
Nox: NADPH oxidase
PN: Postnatal
PW: Pathway
RA: Room air
ROS: Reactive oxygen species
RT-PCR: Real time polymerase chain reaction
S1P: Sphingosine-1-phosphate
S1P_1-5_: S1P receptor 1 through 5
SphK: Sphingosine kinase
WT: Wild type
