# Supplementary material for: Expression profiling of genes regulated by sphingosine kinase1 signaling in a murine model of hyperoxia induced neonatal bronchopulmonary dysplasia
Source: BMC Genomics. 2017 Aug 29;18:664. doi: 10.1186/s12864-017-4048-0 (PMC5576338; doi:10.1186/s12864-017-4048-0)
Supplement: Supplementary file 1 — Sequences of sybr green mouse primers used for RT-PCR. (DOCX 38 kb) [file 12864_2017_4048_MOESM1_ESM.docx]

**Gadd45a**

| **PrimerBank ID** | 6681149a1 |  |  |  |
| --- | --- | --- | --- | --- |
| **Amplicon Size** | 121 |  |  |  |
|  | **Sequence**(5' -> 3') | **Length** | **Tm** | **Location** |
| Forward Primer | CCGAAAGGATGGACACGGTG | 20 | 62.8 | 38-57 |
| Reverse Primer | TTATCGGGGTCTACGTTGAGC | 21 | 61.3 | 158-138 |

**Gadd45g**

| **PrimerBank ID** | 6753938a1 |  |  |  |
| --- | --- | --- | --- | --- |
| **Amplicon Size** | 119 |  |  |  |
|  | **Sequence**(5' −> 3') | **Length** | **Tm** | **Location** |
| Forward Primer | GGGAAAGCACTGCACGAACT | 20 | 63.0 | 67-86 |
| Reverse Primer | AGCACGCAAAAGGTCACATTG | 21 | 62.0 | 185-165 |

**Cdkn1a**

| **PrimerBank ID** | 6671726a1 |  |  |  |
| --- | --- | --- | --- | --- |
| **Amplicon Size** | 103 |  |  |  |
|  | **Sequence**(5' −> 3') | **Length** | **Tm** | **Location** |
| Forward Primer | CCTGGTGATGTCCGACCTG | 19 | 61.7 | 10-28 |
| Reverse Primer | CCATGAGCGCATCGCAATC | 19 | 62.0 | 112-94 |

**Cdkn1b**

| **PrimerBank ID** | | 31542372a1 | |  |  |  |
| --- | --- | --- | --- | --- | --- | --- |
| **Amplicon Size** | | 103 | |  |  |  |
|  | | **Sequence**(5' −> 3') | | **Length** | **Tm** | **Location** |
| Forward Primer | | TCAAACGTGAGAGTGTCTAACG | | 22 | 60.0 | 4-25 |
| Reverse Primer | | CCGGGCCGAAGAGATTTCTG | | 20 | 62.6 | 106-87 |
| **NCBI GeneID** | [**12576**](http://www.ncbi.nih.gov/entrez/query.fcgi?db=gene&cmd=Retrieve&dopt=summary&list_uids=12576) | |  |  |  |  |

**Bax**

| **PrimerBank ID** | | 6680770a1 | |  |  |  |
| --- | --- | --- | --- | --- | --- | --- |
| **Amplicon Size** | | 140 | |  |  |  |
|  | | **Sequence**(5' −> 3') | | **Length** | **Tm** | **Location** |
| Forward Primer | | TGAAGACAGGGGCCTTTTTG | | 20 | 60.2 | 59-78 |
| Reverse Primer | | AATTCGCCGGAGACACTCG | | 19 | 62.1 | 198-180 |
| **NCBI GeneID** | [**12028**](http://www.ncbi.nih.gov/entrez/query.fcgi?db=gene&cmd=Retrieve&dopt=summary&list_uids=12028) | |  |  |  |  |

**Serpine1**

| **PrimerBank ID** | | 6679373a1 | |  |  |  |
| --- | --- | --- | --- | --- | --- | --- |
| **Amplicon Size** | | 116 | |  |  |  |
|  | | **Sequence**(5' −> 3') | | **Length** | **Tm** | **Location** |
| Forward Primer | | TTCAGCCCTTGCTTGCCTC | | 19 | 62.6 | 12-30 |
| Reverse Primer | | ACACTTTTACTCCGAAGTCGGT | | 22 | 61.3 | 127-106 |
| **NCBI GeneID** | [**18787**](http://www.ncbi.nih.gov/entrez/query.fcgi?db=gene&cmd=Retrieve&dopt=summary&list_uids=18787) | |  |  |  |  |

**Serpina3n**

| **PrimerBank ID** | | 6678093a1 | |  |  |  |
| --- | --- | --- | --- | --- | --- | --- |
| **Amplicon Size** | | 116 | |  |  |  |
|  | | **Sequence**(5' −> 3') | | **Length** | **Tm** | **Location** |
| Forward Primer | | ATTTGTCCCAATGTCTGCGAA | | 21 | 60.2 | 1131-1151 |
| Reverse Primer | | TGGCTATCTTGGCTATAAAGGGG | | 23 | 61.3 | 1246-1224 |
| **NCBI GeneID** | [**20716**](http://www.ncbi.nih.gov/entrez/query.fcgi?db=gene&cmd=Retrieve&dopt=summary&list_uids=20716) | |  |  |  |  |

**Saa3**

| **PrimerBank ID** | | 6755396a1 | |  |  |  |
| --- | --- | --- | --- | --- | --- | --- |
| **Amplicon Size** | | 248 | |  |  |  |
|  | | **Sequence**(5' −> 3') | | **Length** | **Tm** | **Location** |
| Forward Primer | | TGCCATCATTCTTTGCATCTTGA | | 23 | 60.8 | 15-37 |
| Reverse Primer | | CCGTGAACTTCTGAACAGCCT | | 21 | 62.3 | 262-242 |
| **NCBI GeneID** | [**20210**](http://www.ncbi.nih.gov/entrez/query.fcgi?db=gene&cmd=Retrieve&dopt=summary&list_uids=20210) | |  |  |  |  |

**Bmp7**

| **PrimerBank ID** | | 31982487a1 | |  |  |  |
| --- | --- | --- | --- | --- | --- | --- |
| **Amplicon Size** | | 164 | |  |  |  |
|  | | **Sequence**(5' −> 3') | | **Length** | **Tm** | **Location** |
| Forward Primer | | ACGGACAGGGCTTCTCCTAC | | 20 | 62.8 | 293-312 |
| Reverse Primer | | ATGGTGGTATCGAGGGTGGAA | | 21 | 62.4 | 456-436 |
| **NCBI GeneID** | [**12162**](http://www.ncbi.nih.gov/entrez/query.fcgi?db=gene&cmd=Retrieve&dopt=summary&list_uids=12162) | |  |  |  |  |

**Wnt5a**

| **PrimerBank ID** | | 6678597a1 | |  |  |  |
| --- | --- | --- | --- | --- | --- | --- |
| **Amplicon Size** | | 128 | |  |  |  |
|  | | **Sequence**(5' −> 3') | | **Length** | **Tm** | **Location** |
| Forward Primer | | CAACTGGCAGGACTTTCTCAA | | 21 | 60.2 | 211-231 |
| Reverse Primer | | CATCTCCGATGCCGGAACT | | 19 | 61.5 | 338-320 |
| **NCBI GeneID** | [**22418**](http://www.ncbi.nih.gov/entrez/query.fcgi?db=gene&cmd=Retrieve&dopt=summary&list_uids=22418) | |  |  |  |  |

**Cdh2**

| **PrimerBank ID** | | 6680902a1 | |  |  |  |
| --- | --- | --- | --- | --- | --- | --- |
| **Amplicon Size** | | 101 | |  |  |  |
|  | | **Sequence**(5' −> 3') | | **Length** | **Tm** | **Location** |
| Forward Primer | | AGCGCAGTCTTACCGAAGG | | 19 | 61.7 | 124-142 |
| Reverse Primer | | TCGCTGCTTTCATACTGAACTTT | | 23 | 60.5 | 224-202 |
| **NCBI GeneID** | [**12558**](http://www.ncbi.nih.gov/entrez/query.fcgi?db=gene&cmd=Retrieve&dopt=summary&list_uids=12558) | |  |  |  |  |

**Aplnr**

| **PrimerBank ID** | | 6753014a1 | |  |  |  |
| --- | --- | --- | --- | --- | --- | --- |
| **Amplicon Size** | | 174 | |  |  |  |
|  | | **Sequence**(5' −> 3') | | **Length** | **Tm** | **Location** |
| Forward Primer | | GGTTACAACTACTATGGGGCTGA | | 23 | 61.1 | 13-35 |
| Reverse Primer | | AGCTGAGCGTCTCTTTTCGC | | 20 | 62.8 | 186-167 |
| **NCBI GeneID** | [**23796**](http://www.ncbi.nih.gov/entrez/query.fcgi?db=gene&cmd=Retrieve&dopt=summary&list_uids=23796) | |  |  |  |  |

**S1pr1**

| **PrimerBank ID** | | 21687214a1 | |  |  |  |
| --- | --- | --- | --- | --- | --- | --- |
| **Amplicon Size** | | 112 | |  |  |  |
|  | | **Sequence**(5' −> 3') | | **Length** | **Tm** | **Location** |
| Forward Primer | | ATGGTGTCCACTAGCATCCC | | 20 | 61.0 | 1-20 |
| Reverse Primer | | CGATGTTCAACTTGCCTGTGTAG | | 23 | 61.6 | 112-90 |
| **NCBI GeneID** | [**13609**](http://www.ncbi.nih.gov/entrez/query.fcgi?db=gene&cmd=Retrieve&dopt=summary&list_uids=13609) | |  |  |  |  |

**S1pr2**

| **PrimerBank ID** | | 4324651a1 | |  |  |  |
| --- | --- | --- | --- | --- | --- | --- |
| **Amplicon Size** | | 137 | |  |  |  |
|  | | **Sequence**(5' −> 3') | | **Length** | **Tm** | **Location** |
| Forward Primer | | ATGGGCGGCTTATACTCAGAG | | 21 | 61.1 | 1-21 |
| Reverse Primer | | GCGCAGCACAAGATGATGAT | | 20 | 61.1 | 137-118 |
| **NCBI GeneID** | [**14739**](http://www.ncbi.nih.gov/entrez/query.fcgi?db=gene&cmd=Retrieve&dopt=summary&list_uids=14739) | |  |  |  |  |

**S1pr3**

| **PrimerBank ID** | | 6753716a1 | |  |  |  |
| --- | --- | --- | --- | --- | --- | --- |
| **Amplicon Size** | | 120 | |  |  |  |
|  | | **Sequence**(5' −> 3') | | **Length** | **Tm** | **Location** |
| Forward Primer | | ACTCTCCGGGAACATTACGAT | | 21 | 60.4 | 49-69 |
| Reverse Primer | | CAAGACGATGAAGCTACAGGTG | | 22 | 60.7 | 168-147 |
| **NCBI GeneID** | [**13610**](http://www.ncbi.nih.gov/entrez/query.fcgi?db=gene&cmd=Retrieve&dopt=summary&list_uids=13610) | |  |  |  |  |

**Lox**

| **PrimerBank ID** | | 6754568a1 | |  |  |  |
| --- | --- | --- | --- | --- | --- | --- |
| **Amplicon Size** | | 117 | |  |  |  |
|  | | **Sequence**(5' −> 3') | | **Length** | **Tm** | **Location** |
| Forward Primer | | TCTTCTGCTGCGTGACAACC | | 20 | 62.7 | 255-274 |
| Reverse Primer | | GAGAAACCAGCTTGGAACCAG | | 21 | 60.8 | 371-351 |
| **NCBI GeneID** | [**16948**](http://www.ncbi.nih.gov/entrez/query.fcgi?db=gene&cmd=Retrieve&dopt=summary&list_uids=16948) | |  |  |  |  |

**Il6**

| **PrimerBank ID** | | 13624311a1 | |  |  |  |
| --- | --- | --- | --- | --- | --- | --- |
| **Amplicon Size** | | 76 | |  |  |  |
|  | | **Sequence**(5' −> 3') | | **Length** | **Tm** | **Location** |
| Forward Primer | | TAGTCCTTCCTACCCCAATTTCC | | 23 | 60.8 | 488-510 |
| Reverse Primer | | TTGGTCCTTAGCCACTCCTTC | | 21 | 61.1 | 563-543 |
| **NCBI GeneID** | [**16193**](http://www.ncbi.nih.gov/entrez/query.fcgi?db=gene&cmd=Retrieve&dopt=summary&list_uids=16193) | |  |  |  |  |

**Il6ra**

| **PrimerBank ID** | | 52693a1 | |  |  |  |
| --- | --- | --- | --- | --- | --- | --- |
| **Amplicon Size** | | 108 | |  |  |  |
|  | | **Sequence**(5' −> 3') | | **Length** | **Tm** | **Location** |
| Forward Primer | | CCTGAGACTCAAGCAGAAATGG | | 22 | 60.6 | 1152-1173 |
| Reverse Primer | | AGAAGGAAGGTCGGCTTCAGT | | 21 | 62.9 | 1259-1239 |
| **NCBI GeneID** | [**16194**](http://www.ncbi.nih.gov/entrez/query.fcgi?db=gene&cmd=Retrieve&dopt=summary&list_uids=16194) | |  |  |  |  |

**Il6st**

| **PrimerBank ID** | | 6754338a1 | |  |  |  |
| --- | --- | --- | --- | --- | --- | --- |
| **Amplicon Size** | | 180 | |  |  |  |
|  | | **Sequence**(5' −> 3') | | **Length** | **Tm** | **Location** |
| Forward Primer | | CCGTGTGGTTACATCTACCCT | | 21 | 60.9 | 79-99 |
| Reverse Primer | | CGTGGTTCTGTTGATGACAGTG | | 22 | 61.4 | 258-237 |
| **NCBI GeneID** | [**16195**](http://www.ncbi.nih.gov/entrez/query.fcgi?db=gene&cmd=Retrieve&dopt=summary&list_uids=16195) | |  |  |  |  |

**Sphk1**

| **PrimerBank ID** | | 27532969a1 | |  |  |  |
| --- | --- | --- | --- | --- | --- | --- |
| **Amplicon Size** | | 169 | |  |  |  |
|  | | **Sequence**(5' −> 3') | | **Length** | **Tm** | **Location** |
| Forward Primer | | ATGGAACCAGTAGAATGCCCT | | 21 | 60.6 | 1-21 |
| Reverse Primer | | TCCGTTCGGTGAGTATCAGTTTA | | 23 | 60.8 | 169-147 |
| **NCBI GeneID** | [**20698**](http://www.ncbi.nih.gov/entrez/query.fcgi?db=gene&cmd=Retrieve&dopt=summary&list_uids=20698) | |  |  |  |  |

**Sgpl1**

| **PrimerBank ID** | | 31543694a1 | |  |  |  |
| --- | --- | --- | --- | --- | --- | --- |
| **Amplicon Size** | | 114 | |  |  |  |
|  | | **Sequence**(5' −> 3') | | **Length** | **Tm** | **Location** |
| Forward Primer | | CTGAAGGACTTCGAGCCTTATTT | | 23 | 60.0 | 25-47 |
| Reverse Primer | | ACTCCACGCAATGAGCTGC | | 19 | 63.0 | 138-120 |
| **NCBI GeneID** | [**20397**](http://www.ncbi.nih.gov/entrez/query.fcgi?db=gene&cmd=Retrieve&dopt=summary&list_uids=20397) | |  |  |  |  |

**Lpar3**

| **PrimerBank ID** | | 12667796a1 | |  |  |  |
| --- | --- | --- | --- | --- | --- | --- |
| **Amplicon Size** | | 211 | |  |  |  |
|  | | **Sequence**(5' −> 3') | | **Length** | **Tm** | **Location** |
| Forward Primer | | CAAGCGCATGGACTTTTTCTAC | | 22 | 60.1 | 21-42 |
| Reverse Primer | | GAAATCCGCAGCAGCTAAGTT | | 21 | 61.0 | 231-211 |
| **NCBI GeneID** | [**65086**](http://www.ncbi.nih.gov/entrez/query.fcgi?db=gene&cmd=Retrieve&dopt=summary&list_uids=65086) | |  |  |  |  |

| Gene name | Primerbank ID | Forward Primer  5’ to 3’ | Reverse Primer  5’ to 3’ |
| --- | --- | --- | --- |
| *Gadd45a* | 6681149a1 | CCGAAAGGATGGACACGGTG | TTATCGGGGTCTACGTTGAGC |
| *Gadd45g* | 6753938a1 | GGGAAAGCACTGCACGAACT | AGCACGCAAAAGGTCACATTG |
| *Cdkn1a* | 6671726a1 | CCTGGTGATGTCCGACCTG | CCATGAGCGCATCGCAATC |
| *Cdkn1b* | 31542372a1 | TCAAACGTGAGAGTGTCTAACG | CCGGGCCGAAGAGATTTCTG |
| *Bax* | 6680770a1 | TGAAGACAGGGGCCTTTTTG | AATTCGCCGGAGACACTCG |
| *Serpine1* | 6679373a1 | TTCAGCCCTTGCTTGCCTC | ACACTTTTACTCCGAAGTCGGT |
| *Serpina3n* | 6678093a1 | ATTTGTCCCAATGTCTGCGAA | TGGCTATCTTGGCTATAAAGGGG |
| *Saa3* | 6755396a1 | TGCCATCATTCTTTGCATCTTGA | CCGTGAACTTCTGAACAGCCT |
| *Bmp7* | 31982487a1 | ACGGACAGGGCTTCTCCTAC | ATGGTGGTATCGAGGGTGGAA |
| *Wnt5a* | 6678597a1 | CAACTGGCAGGACTTTCTCAA | CATCTCCGATGCCGGAACT |
| *Cdh2* | 6680902a1 | AGCGCAGTCTTACCGAAGG | TCGCTGCTTTCATACTGAACTTT |
| *Aplnr* | 6753014a1 | GGTTACAACTACTATGGGGCTGA | AGCTGAGCGTCTCTTTTCGC |
| *S1pr1* | 21687214a1 | ATGGTGTCCACTAGCATCCC | CGATGTTCAACTTGCCTGTGTAG |
| *S1pr2* | 4324651a1 | ATGGGCGGCTTATACTCAGAG | GCGCAGCACAAGATGATGAT |
| *S1pr3* | 6753716a1 | ACTCTCCGGGAACATTACGAT | CAAGACGATGAAGCTACAGGTG |
| *Lox* | 6754568a1 | TCTTCTGCTGCGTGACAACC | GAGAAACCAGCTTGGAACCAG |
| *Il6* | 13624311a1 | TAGTCCTTCCTACCCCAATTTCC | TTGGTCCTTAGCCACTCCTTC |
| *Il6st* | 6754338a1 | CCGTGTGGTTACATCTACCCT | CGTGGTTCTGTTGATGACAGTG |
| *Il6ra* | 52693a1 | CCTGAGACTCAAGCAGAAATGG | AGAAGGAAGGTCGGCTTCAGT |
| *Sphk1* | 27532969a1 | ATGGAACCAGTAGAATGCCCT | TCCGTTCGGTGAGTATCAGTTTA |
| *Sgpl1* | 31543694a1 | CTGAAGGACTTCGAGCCTTATTT | ACTCCACGCAATGAGCTGC |
| *Lpar3* | 12667796a1 | CAAGCGCATGGACTTTTTCTAC | GAAATCCGCAGCAGCTAAGTT |
| *Chi3l3* | 6753416a1 | CAGGTCTGGCAATTCTTCTGAA | GTCTTGCTCATGTGTGTAAGTGA |
| *Stfa1* | NM_001082543 | GCTGGAGAAAATATCTTCATTAAGA | ATTCATGGCTGAATCTCTTTCCAC |
